# Challenges in the identification of *Chryseobacterium indologenes* and *Elizabethkingia meningoseptica* in cases of nosocomial infections and patients with cystic fibrosis

**DOI:** 10.1016/j.nmni.2017.09.002

**Published:** 2017-09-13

**Authors:** É.B. de Carvalho Filho, F.A.L. Marson, C.E. Levy

**Affiliations:** 1) Department of Pediatrics, School of Medical Sciences, University of Campinas, São Paulo, Brazil; 2) Department of Medical Genetics, School of Medical Sciences, University of Campinas, São Paulo, Brazil; 3) Department of Clinical Pathology, School of Medical Sciences, University of Campinas, São Paulo, Brazil

**Keywords:** Bacterial identification, MALDI-TOF MS, phenotypic methods, Phoenix, VITEK 2

## Abstract

Rare nonfermenting Gram-negative bacilli, such as *Chryseobacterium indologenes* and *Elizabethkingia meningoseptica,* have clinical importance in nosocomial infections and cystic fibrosis (CF), and their identification is a challenge to microbiology laboratories. Thus, the objective of this study was to verify the performance of phenotypic and mass spectrometry (matrix-assisted desorption ionization–time of flight mass spectrometry, MALDI-TOF MS) methods to identify *C. indologenes* and *E. meningoseptica.* In this context, the results obtained with phenotypic methods—namely manual biochemical and automated VITEK 2 (bioMérieux, Marcy l’Etoile, France) and Phoenix tests (Becton Dickinson (BD), San Diego, CA, USA)—and by MALDI-TOF MS—namely MALDI-TOF VITEK MS (MALDI-MS; bioMérieux) and MALDI-TOF BioTyper (MALDI-BD; BD)—of 22 isolates (blood cultures of patients with nosocomial infection (n = 15) and from patients with CF (n = 7)), initially identified as *C. indologenes* and *E. meningoseptica,* were compared. As result, using the manual phenotypic method, it was possible to identify the species level in 18/22; no identification was found in 4/22. There was a low agreement level between manual and VITEK 2 automated phenotypic methods when considering the genus level. The greatest agreement for genus-level identification occurred in MALDI-TOF MS equipment (15/22). When comparing all methods to identify the 22 isolates, there was agreement of 4/22 at the genus level and of 4/22 at the species level. In conclusion, there is low agreement level among identification methods of *C. indologenes* and *E. meningoseptica.* Although MALDI-TOF MS equipment shows a higher agreement level among them, results present low levels of confidence.

## Introduction

Besides being rare, *Chryseobacterium indologenes*, *Elizabethkingia meningoseptica* and nonfermenting bacteria are considered opportunists; they are associated with nosocomial infections and cystic fibrosis (CF) [1], [2], [3], [4], [5]. Because of the lack of knowledge of their existence, as well as difficulty characterizing them and their restricted options for antimicrobial treatment, new diagnostic methods have been studied in a search for greater reliability of identification.

There are a total of 251 articles on *C. indologenes* in the National Center for Biotechnology Information database when the widest possible search criteria are used, including articles on bacterial resistance [6], [7], pneumonia [8], [9], paediatric infections [10], [11] and CF [4], [7]. The same occurs in the case of *E. meningoseptica,* for which the same database lists 1436 published articles concerning contamination of hospital water [12], [13], bacteraemia [14], [15], [16] and CF [4], [17].

The correct identification of the aetiologic agent of infection cases and/or outbreaks, including *C. indologenes* and *E. meningoseptica,* is of fundamental importance for both clinical interpretation and correct choice of antibiotic therapy, also to allowing epidemiologic studies on these microorganisms.

In addition to traditional methods that include the phenotypic methods—which include manual as well as VITEK 2 (bioMérieux, Marcy l’Etoile, France) and Phoenix (Becton Dickinson (BD), San Diego, CA, USA) automated biochemical tests—matrix-assisted desorption ionization–time of flight mass spectrometry (MALDI-TOF MS) has become an important diagnostic resource in microbiologic identification routine. MALDI-TOF MS is an analytical method used to obtain micromolecular weight and structural characteristics of the sample. Adapted to laboratory use, it enables easy and quick diagnosis of several human diseases compared to conventional phenotypic screening and molecular identification methods [18], [19].

The objective of this study was to compare the results obtained with phenotypic techniques (manual, VITEK 2 and Phoenix automated biochemical tests) and MALDI-TOF MS (MALDI-TOF VITEK MS (MALDI-MS; bioMérieux) and MALDI-TOF BioTyper (MALDI-BD; BD)) of 22 isolated strains in blood and respiratory secretions of patients with CF, initially identified as *C. indologenes* and *E. meningoseptica,* in a university center.

## Methods

We conducted a retrospective, cross-sectional and descriptive study of 22 samples of nonfermenting bacteria isolated with a frequency of ≤0.5% in the routine work of a microbiology laboratory of a large public university hospital at the University of Campinas, São Paulo, Brazil. Samples were initially identified as 11 strains of *C. indologenes* and 11 *E. meningoseptica* by using manual and automated phenotypic methods. Samples came from blood cultures of patients with nosocomial infection (*n* = 15) and from patients with CF (*n* = 7). *C. indologenes* (LMG8337) and *E. meningoseptica* (LMG12279) strains standardized by the Belgian Co-ordinated Collections of Micro-organisms LMG Bacteria Collection (BCCM/LMG) were analysed as positive control.

Samples were submitted to the following identification methods: (a) manual phenotypic method, (b) VITEK 2 automated phenotypic method, (c) Phoenix, (d) MALDI-MS and (e) MALDI-BD. In this context, the manual phenotypic methods—VITEK 2 and Phoenix—were performed at least twice.

The manual phenotypic method was conducted by 18 biochemical tests: (carbohydrate metabolism) glucose (C_6_H_12_O_6_) oxidation and fermentation (OF), maltose (C_12_H_22_O_11_) OF, sucrose (C_12_H_22_O_11_) OF, dairy (C_12_H_22_O_11_) OF, Xylose (C_5_H_10_O_5_) OF; (metabolisms of amino acids) via Moeller decarboxylation of lysine (C_6_H_14_N_2_O_2_), ornithine (C_5_H_12_N_2_O_2_), arginine (C_6_H_14_N_4_O_2_), Moeller control base; Simmons citrate agar; aesculin (C_15_H_16_O_9_) hydrolysis; indole production test; 6.5% growth in NaCl; degradation of gelatin; degradation of urea (H_4_N_2_O), DNase test; PYR test (l-pirrolidonil-β-naphthylamide); oxidase test; ONPG (ortho-nitrophenyl-β-d-galactosidase); motility test using blades; resistance to imipenem; and resistance to polymyxin B. Analyses were performed following a published standard protocol [20].

VITEK 2 and Phoenix, and MALDI-BD and MALDI-MS automated phenotypic methods were carried out according to the standard protocol of each modality.

MALDI-MS describes the results at reliability levels by percentage. In this study, we used the following classification: (a) 99.9% to 90%—reliable gender and probable species (green zone); (b) 89.9% to 85%—likely gender (yellow zone); (c) <85%—unreliable result (red zone). In relation to MALDI-BD, the following classification was used: (a) 3000 to 2300—gender and reliable species (green zone); (ii) 2299 to 2000—reliable gender and probable species (green zone); (c) 1999 to 1700—likely gender (yellow zone); (d) <1699—unreliable (red zone) [21].

## Results

The manual phenotypic method identified 18/22 samples. Among the automated phenotypic methods, Phoenix identified 20/22 at the level of ‘excellent,’ and VITEK 2 identified around 14/22. In MALDI-TOF MS equipment, we noted a certain lack of reliability during the process, and an ‘excellent’ identification was obtained in 7/22 of MALDI-MS and 3/22 of MALDI-BD (Table 1).

**Table 1:** Frequency of bacteria samples identified by VITEK 2, Phoenix, MALDI-MS and MALDI-BD according to identification score

Characteristic	*n*
VITEK 2—Quality of identification	
Excellent	14
Very good	4
Good	2
Low discrimination	2
MALDI-MS—Identification score	
Reliable genus and probable species (99.9% to 90%)	7
Identification of probable genus (89.9% to 85%)	3
Unreliable identification (<85%)	6
Not identified	6
MALDI-BD—Identification score II	
Highly probable species	3
Secure genus and probable species identification	10
Probable genus	6
Unreliable	3
Phoenix—Quality of identification	
100% to 96%	20
95% to 93%	1
92% to 90%	1
Total	22

MALDI-BD, MALDI-TOF BioTyper (Becton Dickinson (BD), San Diego, CA, USA); MALDI-MS, MALDI-TOF VITEK MS (bioMérieux, Marcy l’Étoile, France); MALDI-TOF, matrix-assisted laser desorption/ionization time-of-flight mass spectrometry; Phoenix (BD); VITEK 2, VITEK 2 automated phenotypic method (bioMérieux).

The complete data from the bacteria identified by VITEK 2, Phoenix, MALDI-MS and MALDI-BD tools regarding the control strains result from the first identification at the microbiology laboratory of the hospital; sample sources and identification scores are provided in Table 2.

**Table 2:** Bacteria identified by VITEK 2, Phoenix, MALDI-MS and MALDI-BD regarding control strain, first identification, sample source and identification score

Sample	First identification	Source	Phenotypic manual	VITEK 2		Phoenix		MALDI-MS		MALDI-BD	
				Species	QI (%)	Species	QI (%)	Species	Score (%)	Species	Score
1	*Elizabethkingia meningoseptica*	Haemoculture	*E. meningoseptica*	*E. meningoseptica*	99	*E. meningoseptica*	99	*E. meningoseptica*	99	*E. meningoseptica*	1885
2	*Chryseobacterium indologenes*	Haemoculture	*C. indologenes*	*C. indologenes*	99	*Stenotrophomonas maltophilia*	99	*S. maltophilia*	98.5	*S. maltophilia*	2139
3	*E. meningoseptica*	Haemoculture	*C. indologenes*	*E. meningoseptica*	99	*C. indologenes*	99	*E. meningoseptica*	982	*Elizabethkingia miricola*	1994
4	*C. indologenes*	Haemoculture	*E. meningoseptica*	*C. indologenes*	96	*C. indologenes*	98	Not identified		*Chryseobacterium gleum*	2351
5	*C. indologenes*	Haemoculture	*C. indologenes*	*C. indologenes*	99	*C. indologenes*	97	Not identified		*C. indologenes*	212
6	*C. indologenes*	Haemoculture	*C. indologenes*	*C. indologenes*	96	*C. indologenes*	98	*Chryseobacterium* sp.	81.7	*C. gleum*	2061
7	*C. indologenes*	Haemoculture	*C. indologenes*	*C. indologenes*	50	*C. indologenes*	98	*Chryseobacterium* sp.	89.7	*C. indologenes*	2102
8	*C. indologenes*	Haemoculture	*C. indologenes*	*C. indologenes*	99	*C. indologenes*	98	*Chryseobacterium* sp.	85.9	*C. indologenes*	2325
9	*C. indologenes*	Haemoculture	*C. indologenes*	*C. indologenes*	99	*C. indologenes*	97	Not identified		*C. gleum*	1299
10	*C. indologenes*	Haemoculture	*E. meningoseptica*	*E. meningoseptica*	93	*C. indologenes*	99	*C. indologenes*	84	*C. indologenes*	2206
11	*E. meningoseptica*	Haemoculture	*E. meningoseptica*	*E. meningoseptica*	99	*E. meningoseptica*	90	*E. meningoseptica*	75.9	*E. meningoseptica*	2201
12	*E. meningoseptica*	CF	*E. meningoseptica*	*E. meningoseptica*	99	*E. meningoseptica*	99	*E. meningoseptica*	87.6	*E. miricola*	216
13	*E. meningoseptica*	CF	*E. meningoseptica*	*E. meningoseptica*	99	*C. indologenes*	98	*E. meningoseptica*	67.9	*E. miricola*	2005
14	*E. meningoseptica*	CF	*E. meningoseptica*	*E. meningoseptica*	99	*E. meningoseptica*	99	*E. meningoseptica*	99.9	*E. meningoseptica*	1989
15	*C. indologenes*	CF	Inconclusive	*Acinetobacter baumannii*	91	*Burkholderia cepacia*	98	*Burkholderia vietnamiensis*	84.3	*B. vietnamiensis*	2433
16	*E. meningoseptica*	CF	Inconclusive	*A. baumannii*	91	*B. cepacia*	95	*E. meningoseptica*	99.9	*E. miricola*	2093
17	*C. indologenes*	CF	*Sphingomonas paucimobilis*	*S. paucimobilis*	93	*Sphingobacterium spiritivorum*	99	Not identified		*Chryseobacterium* sp.	1638
18	*E. meningoseptica*	CF	Inconclusive	*E. meningoseptica*	50	*E. meningoseptica*	99	Not identified		*E. miricola*	2032
19	*E. meningoseptica*	Haemoculture	*Ralstonia pickettii*	*C. indologenes*	95	*C. indologenes*	99	*E. meningoseptica*	98.2	*E. miricola*	1787
20	*C. indologenes*	Haemoculture	Inconclusive	*Brevundimonas diminnuta*	99	*S. spiritivorum*	99	Not identified		*Chryseobacterium* sp.	1729
21	*E. meningoseptica*	Haemoculture	*E. meningoseptica*	*E. meningoseptica*	99	*E. meningoseptica*	96	*E. meningoseptica*	99.9	*E. meningoseptica*	1758
22	*E. meningoseptica*	Haemoculture	*E. meningoseptica*	*E. meningoseptica*	95	Not identified		*Pseudoxanthomonas kaohsiungensis*	41.8	*S. maltophilia*	1311
LMG8337	*C. indologenes*	BCCM/LMG	*C. indologenes*	*C. indologenes*	94	*C. indologenes*	98	*C. indologenes*	86.3	*C. indologenes*	213
LMG12279	*E. meningoseptica*	BCCM/LMG	*E. meningoseptica*	*E. meningoseptica*	96	*E. meningoseptica*	99	*E. meningoseptica*	78.6	*E. meningoseptica*	198

BCCM/LMG, Belgian Co-ordinated Collections of Micro-organisms LMG Bacteria Collection; CF, cystic fibrosis; MALDI-BD, MALDI-TOF BioTyper (Becton Dickinson (BD), San Diego, CA, USA); MALDI-MS, MALDI-TOF VITEK MS (bioMérieux, Marcy l’Étoile, France); MALDI-TOF, matrix-assisted laser desorption/ionization time-of-flight mass spectrometry; Phoenix (BD); QI, quality of identification; VITEK 2, VITEK 2 automated phenotypic method (bioMérieux).

We observed broader agreement between manual phenotypic method and VITEK 2 with 14/22 at species level, Phoenix and VITEK 2 with 13/22, and in Phoenix and manual phenotypic method, with 11/22 (Table 3). Other comparisons among methods showed a similar results among each other (6 to 9/22 identifications at the species level).

**Table 3:** Comparison among phenotypic manual methods and VITEK 2, MALDI-MS, Phoenix and MALDI-BD, according to identification level of 22 samples of bacteria (*Chryseobacterium indologenes* and *Elizabethkingia meningoseptica*)

Identification level	Manual + VITEK 2	Manual + MALDI-MS	Manual + MALDI-BD	VITEK 2 + MALDI-MS	VITEK 2 + MALDI-BD	MALDI-MS + MALDI-BD	Manual + Phoenix	VITEK 2 + Phoenix	Phoenix + MALDI-MS	Phoenix + MALDI-BD
Genus/species	14	6	7	7	7	6	11	13	7	9
Genus		3	4	3	7	9	1	1	4	6
Discrepancy	4	5	7	6	8	1	5	7	4	6
Inconclusive	4	8	4	6		6	5	1	7	1
Total	22	22	22	22	22	22	22	22	22	22

Data are shown as number of samples.

MALDI-BD, MALDI-TOF BioTyper (Becton Dickinson (BD), San Diego, CA, USA); MALDI-MS, MALDI-TOF VITEK MS (bioMérieux, Marcy l’Étoile, France); MALDI-TOF, matrix-assisted laser desorption/ionization time-of-flight mass spectrometry; Phoenix (BD); VITEK 2, VITEK 2 automated phenotypic method (bioMérieux).

We observe that 4/22 identifications were in green areas—the most reliable ones—of MALDI-MS (99.9% to 90%) and VITEK 2 (excellent) methods (Table 4). However, we found the same number (4/22 inconclusive identifications using MALDI-MS) (inconclusive) with identification of VITEK 2 dark green zone (excellent). For the same result, VITEK 2 (14/22 identifications with excellent result) shows more reliability than MALDI-MS (7/12 identifications with 99.9% to 90% score). In addition, a high number of discordant identifications among the methods (12/22) was observed.

**Table 4:** Comparison between confidence scores of MALDI-MS and VITEK 2 methods and identification levels of 22 samples of bacteria (*Chryseobacterium indologenes* and *Elizabethkingia meningoseptica*)

Identification level	MALDI-MS score										
	99.9% to 90%			89.9% to 85%		<85%			Inconclusive		
	VITEK 2 score			VITEK 2 score		VITEK 2 score			VITEK 2 score		
	Ex	VG	Good	Ex	LDis	Ex	VG	Good	Ex	VG	LDis
Genus/species	4			1		2					
Genus				1	1	1					
Discrepancy	1	1	1				2	1	4	1	1
Total	5	1	1	2	1	3	2	1	4	1	1

Data are shown as number of samples.

Ex, excellent; INC, inconclusive; LDis, low discrimination; MALDI-MS, MALDI-TOF VITEK MS (bioMérieux, Marcy l’Étoile, France); MALDI-TOF, matrix-assisted laser desorption/ionization time-of-flight mass spectrometry; VG, very good; VITEK 2, VITEK 2 automated phenotypic method (bioMérieux).

Table 5 presents the comparisons between levels of identification of MALDI-BD and VITEK 2. There was also a higher number of identifications in the VITEK 2 light green zone (14/22 identifications with a result of ‘excellent’) and an index of discordant identifications (8/22) among the methods.

**Table 5:** Comparison between confidence scores of MALDI-BD and VITEK 2 methods and identification level of 22 samples of bacteria (*Chryseobacterium indologenes* and *Elizabethkingia meningoseptica*)

Identification level	MALDI-BD score									
	3000 to 2300		2299 to 2000				1999 to 1700		1699 to 0	
	VITEK 2 score		VITEK 2 score				VITEK 2 score		VITEK 2 score	
	Ex	Good	Ex	VG	Good	LDis	Ex	VG	Ex	VG
Genus/species	1		2			1	3			
Genus	1		3			1	1		1	
Discrepancy		1	1	1	1		1	1		2
Total	2	1	6	1	1	2	5	1	1	2

Data are shown as number of samples.

Ex, excellent; INC, inconclusive; LDis, low discrimination; MALDI-BD, MALDI-TOF BioTyper (Becton Dickinson (BD), San Diego, CA, USA); MALDI-TOF, matrix-assisted laser desorption/ionization time-of-flight mass spectrometry; VG, very good; VITEK 2, VITEK 2 automated phenotypic method (bioMérieux, Marcy l’Etoile, France).

Table 6 provides comparisons among levels of identification of MALDI-TOF MS, showing heterogeneity at the identification levels of each piece of equipment. We found no high levels of disagreement in identifications (1/22 discrepancy result (MALDI-BD between 1699 to 0 and MALDI-MS <85%) and 6/22 samples identified by MALDI-BD and inconclusive by MALDI-MS).

**Table 6:** Comparison between confidence scores of MALDI-BD and MALDI-MS methods and identification methods of 22 samples of bacteria (*Chryseobacterium indologenes* and *Elizabethkingia meningoseptica*)

Identification level	MALDI-BD score										
	3000 to 2300			2299 to 2000				1999 to 1700		1699 to 0	
	MALDI-MS score			MALDI-MS score				MALDI-MS score		MALDI-MS score	
	89.9% to 85%	<85%	INC	99.9% to 90%	89.9% to 85%	<85%	INC	99.9% to 90%	INC	<85%	INC
Genus/species		1		1		2		3			
Genus	1			1	2	2		2			
Discrepancy										1	
Other^a^			1				2		1		2
Total	1	1	1	2	2	4	2	5	1	1	2

INC, inconclusive; MALDI-BD, MALDI-TOF BioTyper (Becton Dickinson (BD), San Diego, CA, USA); MALDI-MS, MALDI-TOF VITEK MS (bioMérieux, Marcy l’Étoile, France); MALDI-TOF, matrix-assisted laser desorption/ionization time-of-flight mass spectrometry.

a Identified by MALDI-BD and inconclusive by MALDI-MS.

## Discussion

We found disagreement in relation to the identification of *C. indologenes* and *E. meningoseptica* regarding the comparison among the different methods.

Microorganisms such as *E. meningoseptica* and *C. indologenes* are part of the VITEK 2 database. However, bacteria show great similarity among each other, considering that they used to be part of the same genus, *Chryseobacterium* [22]. Positive evidence of indole and resistance to polymyxin characterizes both species, distinguished by positive evidence of DNase in *E. meningoseptica* and negative evidence in *C. indologenes*. Automation via VITEK 2 does not show evidence of DNase and polymyxin resistance, but it is capable of distinguishing the species from the highest number of biochemical tests.

The number of unidentified samples when using the manual phenotypic method (4/22) can be justified by the difficulty and unreliability of reading and interpretation the evidence, such as the verification of motility and oxidase, both of which are essential to define genus/species. Because of the slow metabolism of nonfermenting bacteria, some evidence may suffer from alterations after 3 days or more of incubation.

Among the automated equipment available on the market, we studied VITEK 2 and Phoenix. VITEK 2 has 153 species in its database among *Enterobacteriaceae* and rare nonfermenting bacteria [23]. Even with the available database, VITEK 2 is considered a limiting method because it is used for phenotypic identification, which depends on the growth of the microorganism, the process of which often does not occur in a 24-hour period of incubation—the time limit for completion of analysis. In addition, VITEK 2 does not have evidence for identification based on e.g. motility, DNase and oxidase. Particularly in our study, VITEK 2 presented 2/22 results with identification qualified by equipment in the red zone.

When comparing VITEK 2 and Phoenix, we observed a discrepancy in results in which 13/22 agreed at species level against 7/22 that disagreed. Despite being similar, we observed a considerable difference in the identification levels of *C. indologenes* and *E. meningoseptica.*

In our study, MALDI-MS was able to identify 10/22 samples at the genus/species level, with 6/22 being unreliable and 6/22 not identified, whereas MALDI-BD identified 13/22 at the green zone (total for reliable genus/species and probable genus/species), without any undetermined case. Data suggest differences between the equipment used in regard to the reliability standard. This is evident in Table 6, which lists 13 identifications in MALDI-BD light green area divided into four levels of MALDI-MS identification. Data suggest that MALDI-BD, even with more microorganisms than MALDI-MS, either does not have a specific bank for the analysed nonfermenting Gram-negative bacilli or shows equivalence between scores of 3000 and 2300 for MALDI-BD and index of 99% to 90% for MALDI-MS. Thus, despite being the same method, their database and/or software analysis are different.

Regarding 16S rDNA sequencing, we did not perform the technique. However, in our data, the first identification showed no association with the tools used, and the concordance with the tools for *C. indologenes* was 6/11, and was 4/11 and 7/11 for the manual phenotypic methods, MALDI-BD and VITEK 2, respectively. In addition, only 3/11 samples showed the same result in all tests. These data are in accordance with Souza et al. [24], who compared the results of the same tools with 16S rDNA sequencing in CF. In their study on *C. indologenes* and other uncommon glucose nonfermenting Gram-negative bacteria, the authors observed little agreement between each tool and 16S rDNA sequencing. The same discordance was observed by Chang et al. [25], who performed 16S rDNA sequencing with the following results: 1/40 *C. indologenes* (identified as *E. meningoseptica* by VITEK 2 with low discrimination) and 39/40 *E. meningoseptica* (identified as 36/39 *E. meningoseptica* (33/36 with excellent discrimination and 3/36 with low discrimination), 2/39 as *C. indologenes* (excellent discrimination) and 1/39 as *Stenotrophomonas maltophilia* (excellent discrimination)) by VITEK 2 [25].

This study highlights the difficulties in diagnosing uncommon nonfermenting bacteria, and it shows how the currently available methods are not very reliable, as there is little agreement among them. Hence, there is a need for standardizing the most reliable and feasible identification methods for the microorganisms under analysis.

The importance of correct microbiologic identification reflects on the adequate treatment of diseases caused by them, mainly because it mostly affects immunologically compromised patients [14].

Regarding the limitations of the study, we are aware of that the sensitivity, specificity and accuracy of the different methods that we used may only be fully evaluated using identification by sequencing, which is expensive and complex, and which involves the use of different sequences that allow for better discrimination of genera and species of rare nonfermenting bacteria. Another limitation is the absence of 16S rDNA gene sequences used to study bacterial phylogeny and taxonomy.

In conclusion, analysis by manual phenotypic methods and by VITEK 2, Phoenix, MALDI-MS and MALDI-BD resulted in little agreement at the genus and species level. MALDI-TOF MS methods have an excellent correlation among them in classification of identifications, but they are discordant at confidence-level results. The MALDI-TOF MS method is a promising resource in clinical microbiology that needs to expand its data to be able to discriminate infrequently occurring nonfermenting bacteria.

## Conflict of Interest

None declared.
